# Supplementary material for: The analysis of mitochondrial genome and new distribution of the invasive pest, Leptoglossus occidentalis (Heidemann, 1910)
Source: Mitochondrial DNA B Resour. 2026 May 12;11(6):739–43. doi: 10.1080/23802359.2026.2670064 (PMC13169440; doi:10.1080/23802359.2026.2670064)
Supplement: Leptoglossus_occidentalis_marked_clean.docx [file TMDN_A_2670064_SM9686.docx]

The analysis of mitochondrial genome and new distribution of the invasive pest, *Leptoglossus occidentalis* (Heidemann, 1910)

Wei-Qiong Li^a^, Yue-Shuo Liu^a^, Zhi-Peng Chen^a^, Jin-Li Li^a^, Hong-Xia Hou^b, c^, Guo-Hao Zu^a, c^

^a^College of Horticulture and Landscape, Tianjin Agricultural University, Tianjin, 300392, P. R. China

^b^College of Chemical Engineering and Biotechnology, Xingtai University, Xingtai, Hebei, 054000, P. R. China

^c^Corresponding authors: Hong-Xia Hou [(houhongxia003@126.com)](mailto:(houhongxia003@126.com)), Guo-Hao Zu [(zuguohao@tjau.edu.cn)](mailto:(zuguohao@tjau.edu.cn))

**Abstract**

*Leptoglossus occidentalis* (Heidemann, 1910) (‌Hemiptera: Coreidae) is an invasive species that has invaded China. This study reports the first sequencing and assembly of its complete mitochondrial genome. The results show that the mitochondrial genome of *L. occidentalis* contains 13 protein-coding genes, 22 transfer RNA genes, 2 ribosomal RNA genes, and a non-coding control region, with a total length of 15752 bp and an A+T content of 72.9 %. Based on phylogenetic analyses, *L. occidentalis* and *L.* *membranaceus* (Fabricius, 1781) are sister species. Additionally, in Tianjin, Shaanxi, and Guangdong Provinces are new distribution records of this species documented in this study.

**Keywords**

*Leptoglossus occidentalis*; invasive pests; mitochondrial genome; phylogenetic analysis

**Introduction**

*Leptoglossus occidentalis* (Heidemann, 1910) (Fig.1) (Hemiptera: Coreidae), is an important invasive pest , it originated from western North America and has spread eastward (Zhao 2015, Xu 2014). In 2010, it was first intercepted at Tianjin port in China (Zhu 2010). Since then, Chinese customs authorities have intercepted it multiple times (Xu 2014); however, the colonization of *L. occidentalis* in China has not been confirmed. *L. occidentalis* was discovered on *Pinus thunbergiana* (Parl.) (Filippo Parlatore, 1868) Shandong, marking its first reported occurrence and confirming its colonization in China (Ma 2023, Huang 2023). *L. occidentalis* primarily infests plants of *Pinus* spp. (Pinaceae), with up to 40 different host species, both the adult and nymph stages of *L. occidentalis* damage young pine cones by extracting their juices, leading to fruit drop and causing significant economic losses (Roversi 2011, Loewe-Muñoz 2021, Xu 2014). This study reports the first sequencing and assembly of *L. occidentalis* complete mitochondrial genome, and documents new distribution records of this species in Tianjin, Shaanxi, and Guangdong Provinces, China. The mitogenome’s distinct genetic signatures enable rapid and accurate species identification—critical for detecting its invasive spread, as it is morphologically similar to other bug species. The complete mitogenome and phylogenetic analyses provide a basis for tracing its biogeographic origin, and offer valuable references for formulating targeted control strategies to mitigate its spread and establishment.


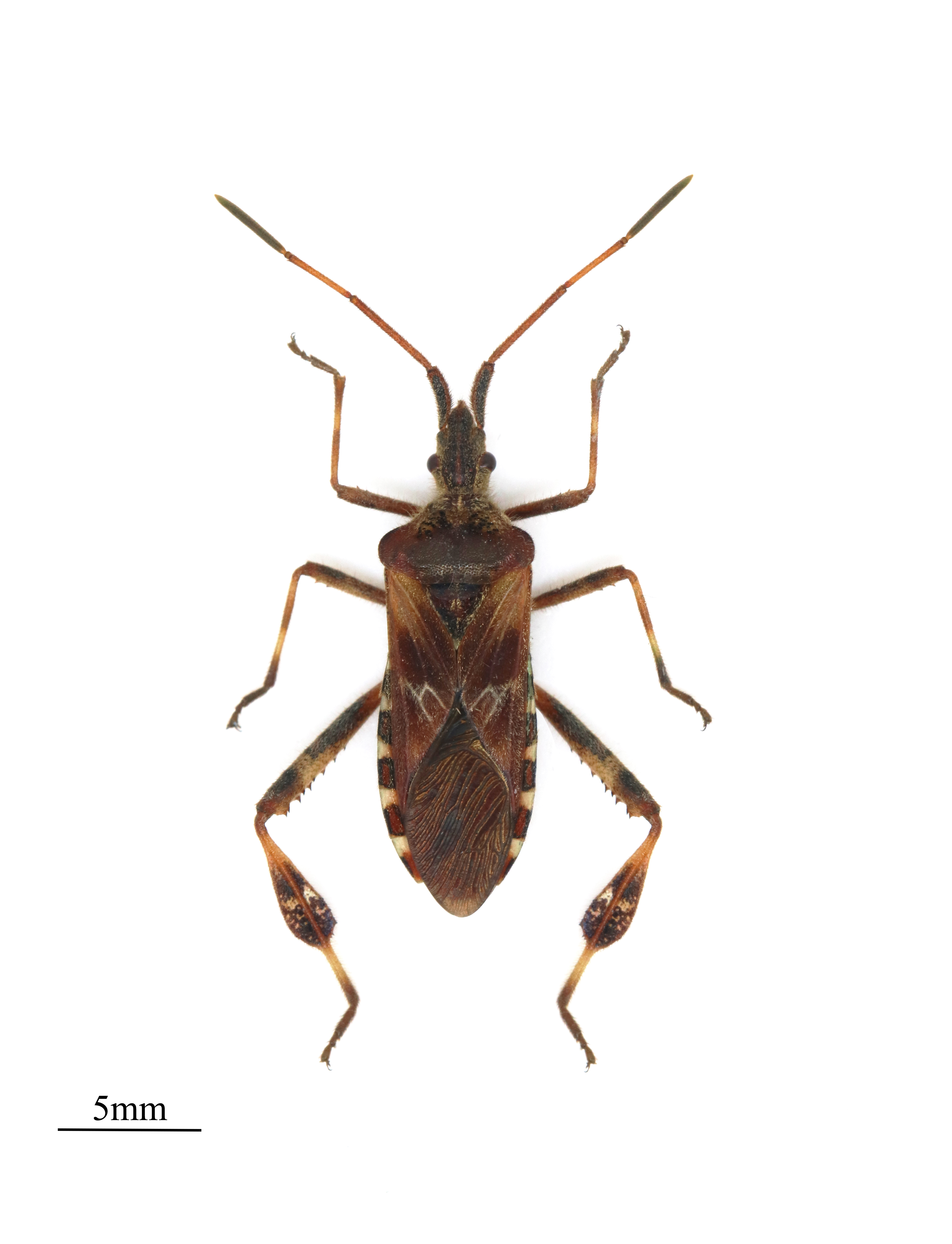


Fig 1. The morphological characteristics of *Leptoglossus occidentalis*. This photo was taken by Guohao Zu at the Entomology Laboratory of Tianjin Agricultural University.

**1 Material and methods**

This study collected seven specimens of *L. occidentalis* for experimentation, of which three (Accession number: TJAU230701-TJAU230703) were collected in June 2023 by Guohao Zu from Jiulong Mountain National Forest Park (117°30′15″E, 40°8′50″N), Jizhou District, Tianjin, one (Accession number: TJAU231001) was collected in October 2023 by Guohao Zu from Tianjin Agricultural University (117°5′38″E, 39°5′21″N), Xiqing District, Tianjin, one (Accession number: TJAU201101) was collected in November 2023 by Jian Liu from Yinghuiwan Community (113°30′16″E, 22°14′20″N), Xiangzhou District, Zhuhai, Guangdong Province, two (Accession number: TJAU241101- TJAU241102) were collected in November 2024 by Mengying Chen from Baota Mountain (109°29′45″E, 36°35′44″N), Yanan City, Shaanxi Province. The specimens were preserved in anhydrous ethanol and stored in a refrigerator at -20℃ before DNA extraction. After being identified based on morphological characters (Ma 2023), genomic DNA from species collected in Jizhou (Accession number: TJAU231001) was extracted using DNeasy Blood & Tissue Kit (Qiagen, Hilden, Germany) on the basis of the manufacturer’s protocol, and the remaining specimens and DNA (Voucher number: Tjau-S2366) deposited at the insect laboratory of Tianjin Agricultural University (https://chl.tjau.edu.cn/, Guohao Zu, zuguohao@tjau.edu.cn).

The Illumina TruSeq library was prepared with an average insert size of 350 bp and sequenced with the paired-end reads length of 150 bp on Illumina NovaSeq 6000 platform (Biomarker Technologies Corporation, Beijing, China). Upon obtaining the raw sequencing data, FastQC software (Andrews 2010) was used for quality assessment to evaluate base quality distribution and adapter contamination. Subsequently, fastp software (Chen et al. 2018) was employed for quality control, specifically including the removal of adapter sequences, trimming of low-quality base ends, and filtering of overly short reads, ultimately yielding high-quality clean data. The Clean data were used to assemble a complete mitogenome using MitoZ v3.6 software (Meng et al. 2019). We mapped the clean data onto the mitochondrial genome to obtain the sequencing coverage depth (Fig. S1).

The gene sequences were preliminarily annotated by MITOS2 (Afgan et al. 2016) on the online platform Galaxy (https://usegalaxy.eu/). The following specific parameters were set: Genetic code: invertebrate (5); Reference data: RefSeq 89 Metazoa, with other settings kept as default. In addition, we obtained five sequences of other Coreidae species that showed the highest similarity to the *L. occidentalis* sequence. The species with the highest similarity to *L. occidentalis* sequence were *L. membranaceus* (Fabricius, 1781) NC042809, *Anoplocnemis curvipes* (Fabricius, 1781) NC035509, *Pseudomictis brevicornis* Hsiao, 1963 NC042814, *Mictis tenebrosa* (Fabricius, 1787) NC042811, and *Cletomorpha* *raja* Distant, 1901 NC063143. These sequences were obtained from the NCBI website (https://www.ncbi.nlm.nih.gov) and manually annotated by comparing the preliminary annotation results with similar sequences using Geneious Prime v. 2022.2.2. The secondary structure of rRNAs was predicted by MITOS2. The nucleotide composition of the mitogenome of *L. occidentalis* was analyzed with MEGA11 (Tamura et al. 2021). The codon usage frequency of PCGs in the mitogenome of *L. occidentalis* was computed using PhyloSuite v1.2.3.

All 13 PCGs were extracted from the complete mitochondrial DNA sequences of 18 closely related taxa of Coreidae. One species from Lygaeidae, *Nysius graminicola* (Kolenati, 1845) (GenBank accession number NC_073587), was used as an outgroup (Table S1). To investigate the phylogeny of *Leptoglossus* in Coreidae, the family-level relationships within Coreidae were reconstructed using 13 PCGs with two inference methods (BI and ML). Each protein-coding gene (PCG) was individually aligned via the MAFFT v.7 online service with the L-INS-i strategy, followed by optimization using MACSE (Ranwez et al. 2018). The aligned sequences were trimmed with GBlocks and concatenated into a combined PCG dataset using PhyloSuite v.1.2.3 (Talavera and Castresana 2007, Zhang et al. 2020). The optimal nucleotide substitution model was selected based on the Bayesian information criterion (BIC) using ModelFinder v.2.2.0 (Kalyaanamoorthy et al. 2017). For BI analysis, MrBayes v.3.2.7a was employed with four chains and two independent runs of 2,000,000 generations, with sampling conducted every 1,000 generations. The first 25% of trees were discarded as bun-in, and convergence was confirmed when the average standard deviation of split frequencies was <0.01 and the potential scale reduction factor (PSRF) approached 1.0. ML analysis was implemented in IQ-TREE, v.2.2.0 (Nguyen et al. 2015) with 1,000 bootstrap replicates under the standard bootstrap approximation.

**2 Results and Analysis**

We obtained a complete mitogenome of *L. occidentalis* (GenBank accession number PP097724), which was 15,752 bp in size and included all the 37 genes (13 PCGs, 22 tRNAs and two rRNAs) and a long non-coding region (Fig. 2). The gene arrangement order is the same as that of the hypothetical progenitor of the Hexapoda *Drosophila yakuba* Burla, 1954 (Shao 2003), and no gene rearrangement has occurred.


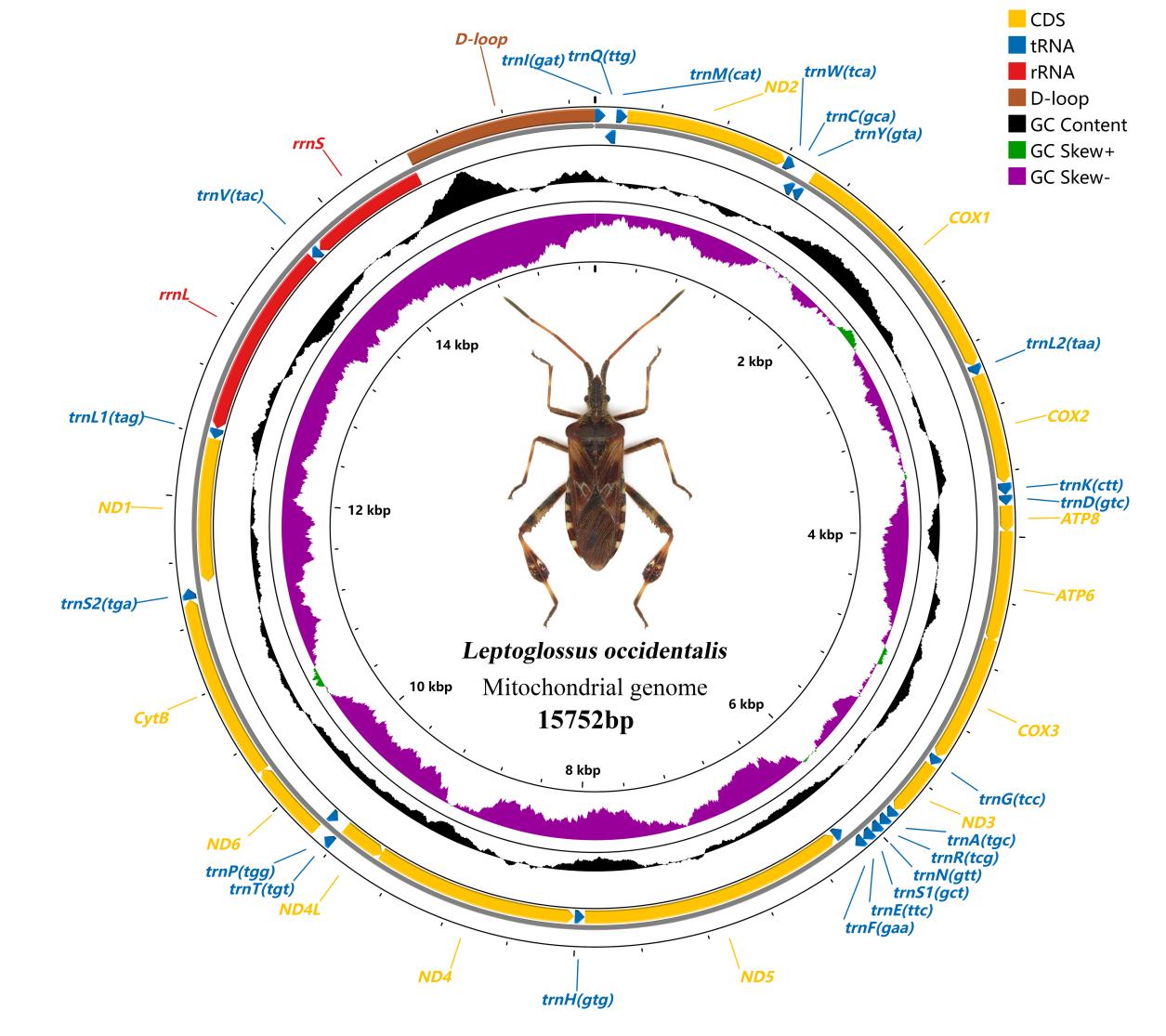


Fig. 2 Gene arrangement in the mitogenome of *L. occidentalis*.

The mitogenome of *L. occidentalis* contains 37 genes, 14 tRNAs and 9 PCGs were encoded on the majority strand (J-strand), while the other 14 genes were encoded on the minority strand (N-strand). The longest gene is ND5, with a length of spanning 1713 bp, while the shortest is *trnA*, spanning 62 bp. The mitogenome of *L. occidentalis*, some of which have gene spacers and over laps with neighbouring genes. 13 gene overlap regions with a total of 32 bp were observed, ranging from 1 to 8 bp in size, and the longest gene overlap region was between *trnW* and *trnC*. Additionally, 8 intergenic regions with a total of 32 bp were observed, ranging from 1 to 21 bp in size, and the longest intergenic region was between *trnS2* and *ND1*. The start codons of all PCGs are “ATN”, except *COX1* uses TTG instead of the usual ATN start, and the complete stop codon TAA and TAG was widely assigned to 9 protein-coding genes, respectively, while *COX1、COX2、ATP6* and *ND1* used a single T or TA residue as an incomplete stop codon (Table S2).

The A+T content of the mitogenome of *L. occidentalis* was 72.9%, and the mitogenome of *L. occidentalis* is biased toward A and T, and an AT-Skew of 0.082, indicating a clear AT preference. Among the 13 PCGs, the AT content of 11 is above 70%, with *ATP8* having the highest AT content at 82.1%. The rRNAs, *rrnS* and *rrnL*, have AT contents of 75.2% and 74.2%, respectively, while the 22 tRNAs have an AT content of 74.3% (Table S3). The codon usage in the mitogenome of *L. occidentalis* indicates that the most frequently used codon is UUA with a relative synonymous codon usage (RSCU) of 3.84, while the least frequently used codons are CCG and CUC with an RSCU of 0.03(Fig. S2).

The mitogenome of *L. occidentalis* contains 22 tRNA sequences ranging from 62 to 74 bp in length. The longest tRNA is *trnK* at 74 bp, and the shortest is *trnA* at 62 bp. All tRNAs except for *trnS1*, which lacks a DHU arm, can be folded into a typical cloverleaf structure. In addition to normal base pairing, there were 23 base mismatches, including 21 common G-U mismatches, a U-U mismatch on the *trnA* acceptor arm, and a C-U mismatch on the TψC arm of *trnV* (Fig. S3).

Phylogenetic analysis (Fig. 3) revealed that the closest relative of *L. occidentalis* is *L. membranaceus*. The phylogenetic analysis is consistent with morphological classification, and *Leptoglossus* demonstrates close phylogenetic affinity to *Mictis*, *Pseudomictis*, *Molipteryx*, and *Notopteryx,* with exceptionally high nodal support values. The phylogenetic tree constructed from the nucleotide sequence data matrix of protein-coding genes, resolving the developmental relationships among 12 genera within the family Coreidae, is consistent with the results of (Tian et al 2023).


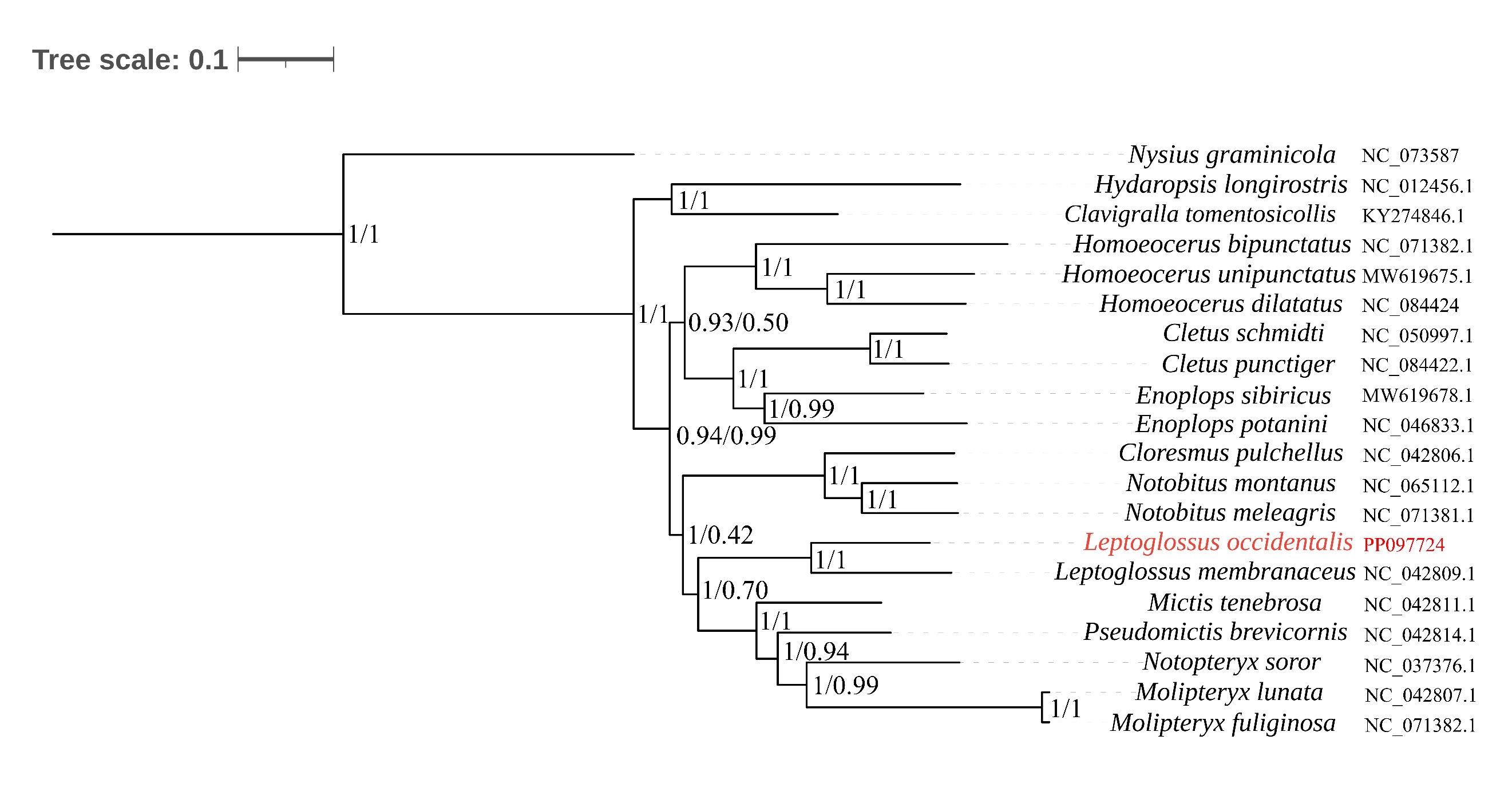


Fig. 3 The phylogenetic tree was constructed based on 13 PCGs by Bayesian inference and maximum likelihood methods. The number at each node indicate the posterior probability and bootstrap values resulting from the analyses. (ML on the left and BI on the right). The species name and corresponding NCBI accession number of each species involved in this phylogenetic tree are shown on the right. The following sequences were used: *Clavigralla tomentosicollis* KY274846 (Liu et al. 2019), *Cletus punctiger* NC_050997 (Diect Submission), *Cletus schmidti* NC_084422 (Liu et al. 2019), *Cloresmus pulchellus* NC_042806 (Diect Submission), *Enoplops potanini* NC_046833 (Zhu et al. 2019), *Enoplops sibiricus* MW619678 (Liu et al. 2019), *Homoeocerus bipunctatu* NC_071382 (Hua et al. 2008), *Homoeocerus dilatatus* NC_084424 (Liu et al. 2019), *Homoeocerus unipunctatus* MW619675 (Tian et al 2021), *Hydaropsis longirostris* NC_012456 (Ye et al. 2022), *Leptoglossus membranaceus* NC_042809 (Zhu et al. 2019), *Mictis tenebrosa* NC_042811 (Zhang et al. 2019), *Molipteryx fuliginosa* NC_084425 (Steele et al. 2017), *Molipteryx lunata* NC_042807 (Ye et al. 2022), *Notobitus meleagris* NC_071381 (Diect Submission), *Notobitus montanus* NC_065112 (Jiang et al. 2017), *Notopteryx soror* NC_037376 (Liu et al. 2019), *Nysius graminicola* NC_073587 (Liu et al. 2019), *Pseudomictis brevicornis* NC_042814 (Lin et al. 2023).

**3 Conclusion**

As a potential exotic invasive pest, *L. occidentalis* has not been previously detected in large populations in China (Wu 2023). However, this study reports the first discovery of *L. occidentalis* in Tianjin, Shaanxi, and Guangdong Provinces. After comparing and analyzing the complete mitochondrial genome of *L. occidentalis* with the complete mitochondrial genome of related species, its phylogenetic placement within the family Coreidae was determined, which provides a foundation for tracing its biogeographical origins and offers valuable references for developing targeted control strategies to mitigate its dispersal and establishment.

**Acknowledgements**

We thank Liu Jian for providing the essential Guangdong Province specimens that greatly enriched this research.

**Author contributions**

Wei-Qiong Li and Yue-Shuo Liu contributed significantly to analysis and manuscript preparation. Guo-Hao Zu, Hong-Xia Hou, Jin-Li Li and Zhi-Peng Chen performed experiments and data analyses. Guo-Hao Zu selected species and took species photos. Guo-Hao Zu and Wei-Qiong Li were involved in the revising paper critically for intellectual content. Guo-Hao Zu contributed to the conception and design of the study and the final approval of the version to be published. All authors agree to be accountable for all aspects of the work.

**Disclosure statement**

No potential conflict of interest was reported by the authors. The authors are responsible for the content and writing of the paper.

**Funding**

This work was supported by Tianjin Basic Research Fund for Scientific Research in Higher Education Institutions (2021KJ115), the National Natural Science Foundation of China (Grant number: 32200375), and the Natural Science Foundation of Hebei Province, China (C2024108006).

**Ethics statement**

*L. occidentalis* used in this study comply with international ethical requirements. Our practices are in full compliance with Chinese legislation, as well as adhering to pertinent guidelines, policies, and regulations. Notably, the collection site is not designated as a nature reserve, and the acquisition of specimens was executed without causing harm to the local ecology. Furthermore, all experiments were carried out under the vigilant supervision and with the explicit permission of Tianjin Agricultural University.

**ORCID**

Guohao Zu <https://orcid.org/0000-0002-9892-2171>

Weiqiong Li https://orcid.org/0009-0004-9067-2149

Yueshuo Liu https://orcid.org/0009-0008-7007-9419

Zhipeng Chen https://orcid.org/ 0009-0006-9947-5074

Jinli Li https://orcid.org/0000-0001-8394-3829

Hongxia Hou https://orcid.org/0000-0002-0203-8809

**Data availability statement**

The genome sequence data that support the findings of this study are openly available in GenBank of NCBI at [https://www.ncbi.nlm.nih.gov] (https://www.ncbi.nlm.nih.gov/) under the accession no. PP097724. The associated **BioProject**, **SRA**, and **Bio-Sample** numbers are PRJNA1289996, SRR34517778, and SAMN49907159 respectively.”

**References**

Zhao L, Zhu GP, Li M. 2015. Potential distribution of *Leptoglossus occidentalis* and *Jadera haematoloma* in China. J. Tianjin Norm Univ (Nat Sci Ed). 35(1): 75–78.

Xu M, Qian L, An YL. 2014. A dangerous pest—the western conifer seed bug, *Leptoglossus occidentalis* Heidemann. Plant Quar. 28(1): 67–71.

Zhu WB. 2010. Exotic Coreid Bugs Introduced into China. In Proceedings of the 4th Meeting of the International Heteropterist's Society. Nankai University, Tianjin.

Ma RY, Pan T, Wang TT. 2023. Morphological and molecular biological Identification of *Leptoglossus occidentalis*. J. West China For Sci. 52(2): 10–17.

Huang ZC, Liu TT. 2023. Tracking the invasive pest *Leptoglossus occidentalis*. Heidemann, 1910 in China. J. Environ. Entomol. 45(5): 1224–1235.

Roversi PF, Strong WB, Caleca V. 2011. Introduction into Italy of *Gryon pennsylvanicum* (Ashmead), an egg parasitoid of the alien invasive bug *Leptoglossus occidentalis* Heidemann. Eppo Bull. 41(1): 72–75. doi: 10.1111/j.1365-2338.2011.02439.x

Loewe-Muñoz V, Claudia D, del Río R. 2021. Western conifer seed bug (*Leptoglossus occidentalis*) challenging stone pine cropping in the Southern Hemisphere. For Ecol Mnag. 496: 119–434. doi:10.1016/j.foreco.2021.119434

Andrews S. (2010). FastQC: a quality control tool for high throughput sequence data (online). http://www.bioinformatics.babraham.ac.uk/projects/fastqc/.

Meng GL, Li YY, Yang CT, Liu SL. 2019. MitoZ: A toolkit for animal mitochondrial genome assembly, annotation and visualization. Nucleic Acids Res. 47(11): e63. doi:10.1093/nar/gkz173.

Afgan E, Baker D, Batut B, van den Beek M, Bouvier D, Cech M, Chilton J, Clements D, Coraor N, Grüning B, Guerler A, Hillman-Jackson J, Hiltemann S, Jalili V, Rasche E, Soranzo N, Turaga N, Taylor J, Nekrutenko A, Goecks J. 2016. The Galaxy platform for accessible, reproducible and collaborative biomedical analyses: 2016 update. Nucleic Acids Res. 44(W1): W3–W10. Doi:10.1093/nar/gkw343.

Tamura K, Stecher G, Kumar S. 2021. MEGA11: Molecular Evolutionary Genetics Analysis version 11. MOL BIOL EVOL. 38(7): 3022–3027. doi:10.1093/molbev/msab120.

Ranwez V, Harispe S, Dufayard JF, Koncz C, Champagnat F, Ponty Y, Lartillot N, Galtier N, Thompson JD. 2018. MACSE v2: Toolkit for the alignment of coding sequences accounting for frameshifts and stop codons. MOL BIOL EVOL. 35(9): 2210–2215. doi:10.1093/molbev/msy159.

Talavera G, Castresana J. 2007. Improvement of phylogenies after removing divergent and ambiguously aligned blocks from protein sequence alignments. Syst Biol. 56(4): 564–577. doi:10.1080/10635150701472164.

Zhang D, Gao FL, Jakovlić I, Zou H, Zhang J, Li WX, Wang GT. 2020. PhyloSuite: An integrated and scalable desktop platform for streamlined molecular sequence data management and evolutionary phylogenetics studies. Mol Ecol Resour. 20(1): 348–355. doi:10.1111/1755-0998.13096.

Kalyaanamoorthy S, Minh BQ, Wong TKF, von Haeseler A, Jermiin LS. 2017. ModelFinder: Fast model selection for accurate phylogenetic estimates. Nat Methods. 14(6): 587–589. doi:10.1038/nmeth.4285.

Nguyen LT, Schmidt HA, von Haeseler A, Minh BQ. 2015. IQ-TREE: A fast and effective stochastic algorithm for estimating maximum-likelihood phylogenies. MOL BIOL EVOL. 32(1):268–274. doi:10.1093/molbev/msu300.

Shao RF, Barker SC. 2003. The highly rearranged mitochondrial genome of the plague thrips, *Thrips imaginis* (Insecta: Thysanoptera): convergence of two novel gene boundaries and an extraordinary arrangement of rRNA genes. Mol Biol Evol. 20(3): 362–370. doi:10.1093/molbev/msg045

Wu XX, Zheng QL, Lei H. 2023. Identification, damage, invasion, and control strategies of *Leptoglossus occidentalis*. Sichuan Agric Sci Technol. 5: 43–46. doi:10.3969/j.issn.1004-1028.2023.05.013.

Tian XK. 2023. Mitochondrial genome of bamboo pest Paradasynus spinosus and phylogenetic analysis of Coreoidea. [Master's thesis]. Guiyang: Guizhou Normal University.
