# Supplementary material for: The analysis of mitochondrial genome and new distribution of the invasive pest, Leptoglossus occidentalis (Heidemann, 1910)
Source: Mitochondrial DNA B Resour. 2026 May 12;11(6):739–43. doi: 10.1080/23802359.2026.2670064 (PMC13169440; doi:10.1080/23802359.2026.2670064)
Supplement: Supplementary_Figure.docx [file TMDN_A_2670064_SM9685.docx]

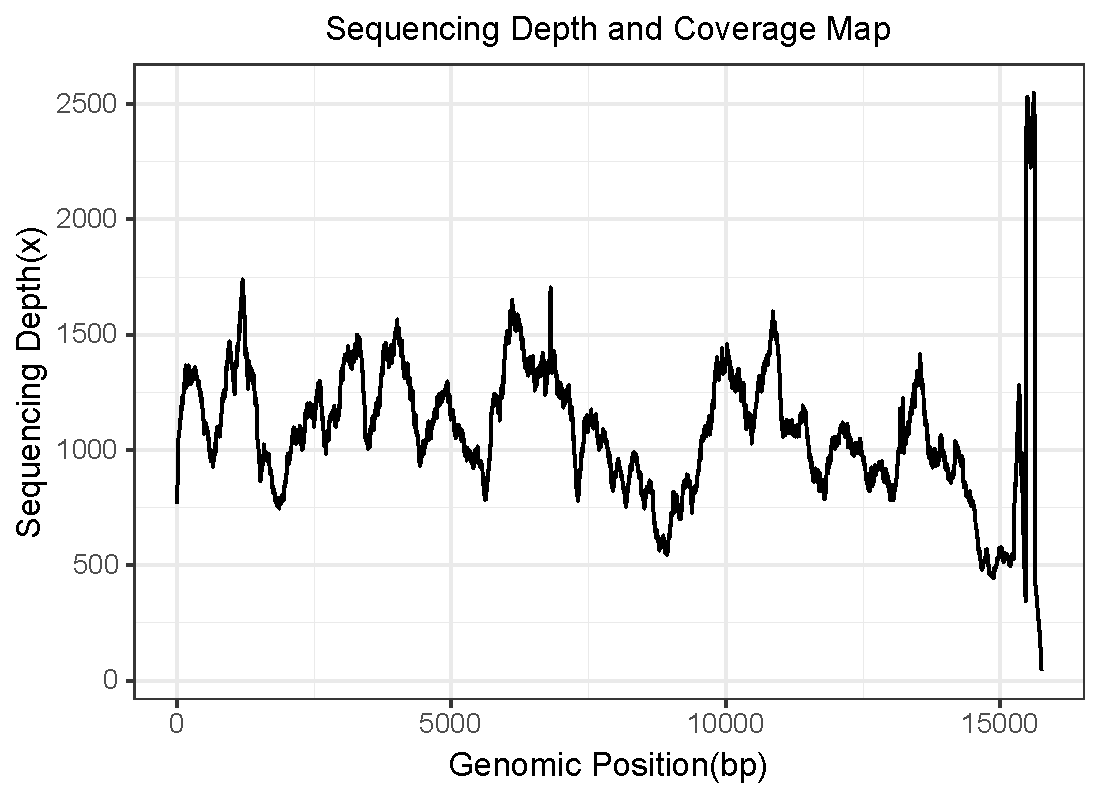


Supplementary Figure. S1 The sequencing coverage depth of *Leptoglossus occidentalis*

*
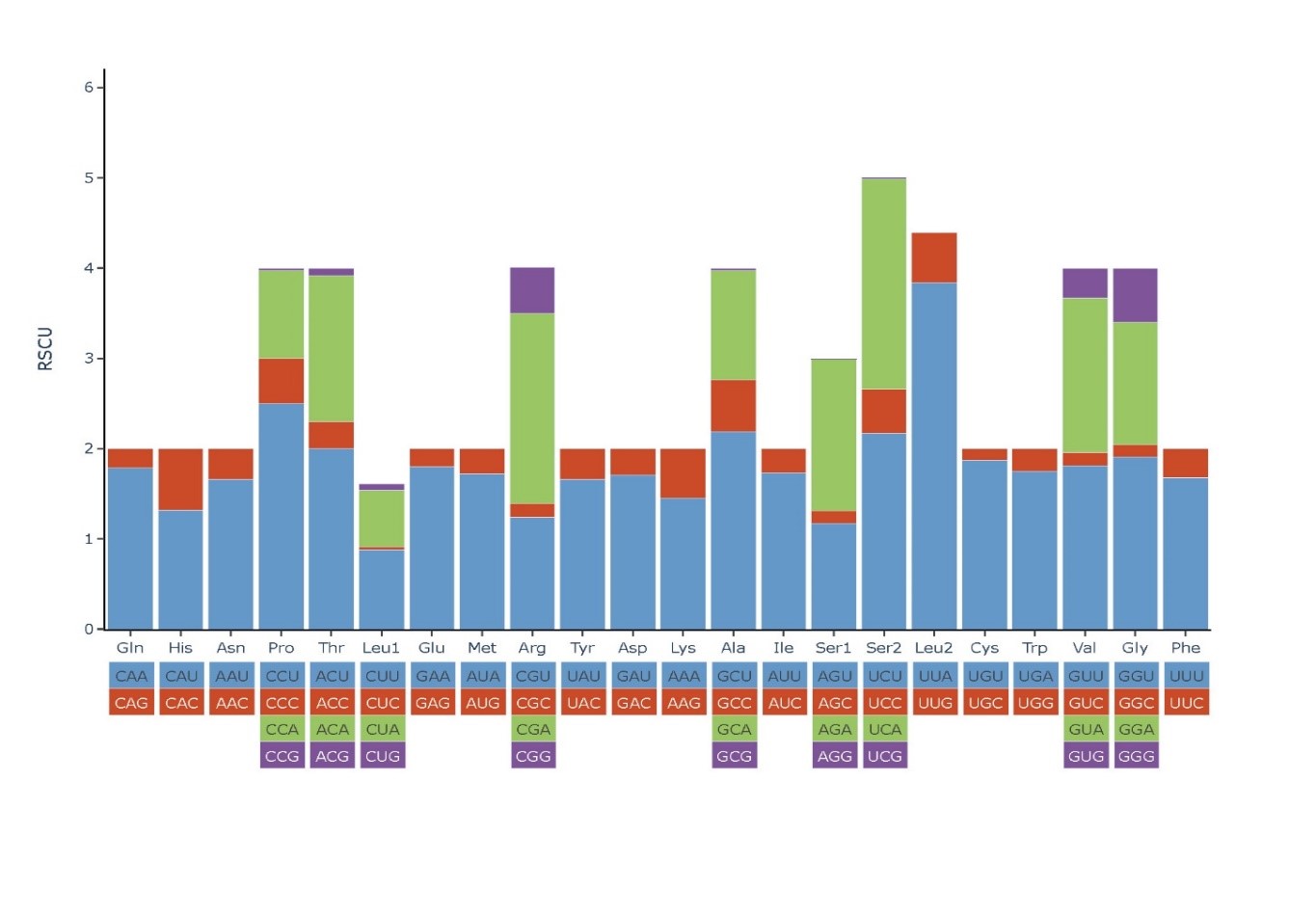
*

Supplementary Figure. S2 Relative synonymous codon usage of *L. occidentalis*


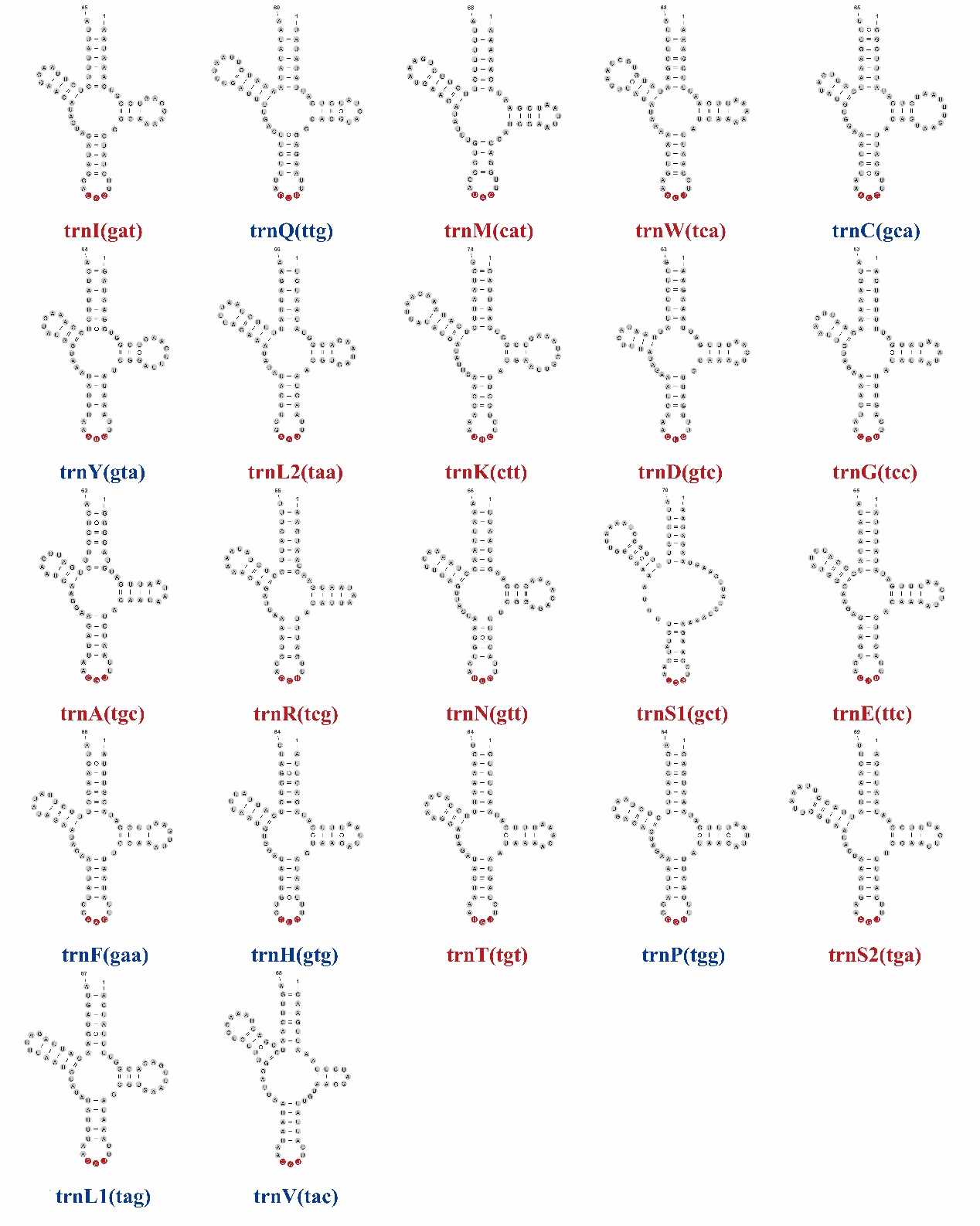


Supplementary Figure. S3 Secondary structure of 22 transfer RNA genes(tRNAs) of *L. occidentalis*
